# Special Issue Editorial: Applications of 3D Printing for Polymers

**DOI:** 10.3390/polym15071638

**Published:** 2023-03-25

**Authors:** Paul F. Egan

**Affiliations:** Mechanical Engineering, Texas Tech University, Lubbock, TX 79409, USA; paul.egan@ttu.edu

Polymer 3D printing is an emerging technology highly relevant in diverse industries, including medicine, electronics, and robotics. Polymer printing has many advantages; polymers possess a wide range of material properties, are inexpensive to process, and enable highly accurate fabrication for complex designs. Over the past decade, research in 3D printing in polymers has grown exponentially (Figure 1), largely due to technological innovations with increased design awareness and accessibility. Although 3D printing technologies have advanced substantially in previous years, there are still many research challenges to address regarding materials, processing, and design. This editorial highlights these challenges by summarizing the “Applications of 3D Printing for Polymers” Special Issue for *Polymers* that includes 16 peer-reviewed articles consisting of 4 review articles and 12 research articles. The original research published includes investigations in specified 3D printing applications, processing parameters and testing, and mechanics and manufacturing characterization.

Review articles covered areas of polymer 3D printing focused on specific topic areas such as vat polymerization and printing polyether ether ketone (PEEK) via fused filament fabrication to broader topics including 3D printing applications in medicine and process optimization for industrial robotics. The review of vat polymerization from Pagac et al. highlighted processes including stereolithography, digital light processing, and continuous digital light processing, aiming to inspire readers to engage in further research such as new materials and mathematical models for microrods and bionic structures [1]. Dua et al.’s review focused on PEEK for extrusion printing processes that highlighted the material’s versatility in medical, aerospace, electrical, and chemical application areas [2]. PEEK is most often used in the medical industry and has advantages in forming patient-specific implants with a higher degree of accuracy than metal printing, while maintaining biocompatibility and providing an elastic modulus similar to human bone. Arefin et al. highlighted the different ways material, process, and design strategies are used for medical applications, with detailed examples provided for the application areas of tissue scaffolds, dental implants, wearable prosthetics, safety equipment, surgical planning, and drug delivery [3]. Lastly, the review of industrial robotics manufacture using polymer-based additive manufacturing conducted by Walia et al. highlighted the complexity of robotic development that requires multiple design iterations [4]. They found that 3D printing provides an inexpensive method for rapid design iterations, and an extensive range of new polymer-based additive techniques and materials are on the horizon to further improve the design and development process. 

A first set of research articles conducted in-depth investigations for applications and case studies, with focuses on personal protective equipment, surgical guides, orodispersible films, and microwave circuits. Rendeki et al. provided a timely article regarding the production of personal protective equipment during the era of the COVID-19 pandemic [5]. They found that compared to production methods such as injection molding, 3D printing reduced the lead time, which also reduced the upfront cost due to the absence of molding requirements. Groove sealing for surgical guides was investigated by Lim et al., who extensively characterized the accuracy of the implant placements and determined that sealing the groove of the tooth prior to manufacturing promotes improved accuracy [6]. Panraksa et al. investigated the feasibility of five different kinds of hydrophilic polymers for orodispersible film by studying their physicochemical and mechanical properties [7]. Stereolithography printing was researched by Torregrosa-Penalva et al. for manufacturing high-frequency circuits [8]. They found that electrical characterization provides a relative dielectric permittivity of 3.25 and a loss tangent of 0.03 for their polymeric resin that informed a proof-of-concept design for a complex geometry stepped impedance filter on a multi-height substrate. 

Research articles continued with investigations focused on the parameters and processing of materials for 3D printing and their effects related to performance in relevant application areas. Effects of postcuring for dental materials was investigated by Bayarsaikhan et al. [9]. Their team tested the mechanics and cell viability of the resin as the postcuring temperatures were altered for different periods of time. Buj-Corral et al. measured how printing parameters affected the surface roughness for fused filament fabrication technology [10]. They used the factorial design of experiments to alter the nozzle diameter, temperature, layer height, print speed, and extrusion multiplier and then used an adaptive neural fuzzy inference system to interpret the results. Pressure orientation-dependent recovery, studied by G. Ehrmann and A. Ehrmann, demonstrated anisotropy in 3D printed parts which informs the ideal orientation of 3D printed structures such as bumpers or orthoses [11]. Low-cost printed polymers investigated by Storck et al. were periodically heat-treated in a manner similar to the temperature cycles experienced by microsatellites in low earth orbit [12]. Several polymers were investigated with respect to their dimensional stability and mechanical properties, with findings suggesting that poly(lactic acid) filaments were the most suitable.

The final set of articles provided empirically grounded studies for characterizing the mechanics and manufacturing of the 3D printing process. Bagalkot et al. investigated the use of 3D printed injection molds as a cost-effective method for low-volume injection molding [13]. They found, through theoretical and experimental grounds, that raised features in molds do not fail by bending failure, but rather by chipping of the edges of raised features. Lattices were studied by Silva et al., who demonstrated fused filament fabrication was able to create diverse open cell structures capable of supporting compressive loads, despite some limitations in quality and accuracy [14]. CAD/CAM milling influences were investigated by Ellakany et al. for an interim fixed dental prosthesis after thermomechanical aging. They found that the milled and stereolithography-printed interim fixed dental prosthesis demonstrated similar mechanical properties for force at break and flexural strength, but stereolithography-printed parts had a lower elastic modulus [15]. Nguyen et al. performed an experimental modal analysis of 3D printed structures [16]. They found enhanced performance in skirt-adhesion-type specimens, which was likely due to the enhanced heat transfer between the 3D printed bed and the specimen during fabrication. These results provide an improved understanding of heat transfer and 3D printing that informs how to better control the processing parameters to improve printed parts for specified performance.

Overall, the collection of articles for this Special Issue provided novel contributions across a spectrum of polymer research topics in 3D printing including materials, processes, designs, and applications. The research addressed many of the important research areas and challenges for polymer 3D printing, while also establishing new routes towards better understanding and incorporating 3D printed polymers to their fullest potential in diverse applications. 

## Figures and Tables

**Figure 1:**
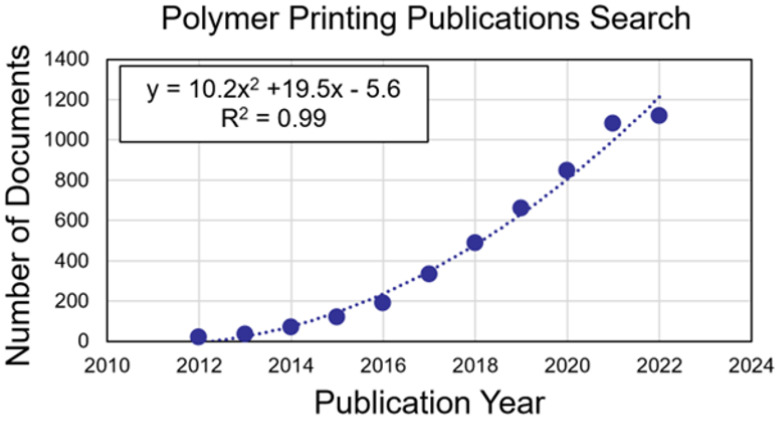
Number of documents found according to Scopus over the past decade for keyword search of: ((“3D Printing” OR “Additive Manufacturing”) AND Polymers).
